# Knowledge Translation Interventions for Youth Health Care Transitions: Protocol for a Realist Review

**DOI:** 10.2196/99731

**Published:** 2026-07-15

**Authors:** Grace MacIntyre, Rachel Flynn, Kathryn A Birnie, Leah Boulos, Sarah M Gallant, Ian D Graham, Amy Grant, Megan Gray, Gillian Harvey, Amanda Higgins, Carla T Hilario, Julia C Kontak, Deborah A McNeil, Sarah Munce, Jacklynn Pidduck, Shannon D Scott, Alene Toulany, Christine E Cassidy

**Affiliations:** 1 School of Nursing, Faculty of Health, Dalhousie University, 5850/5980 University Avenue P.O. Box 9700, Room K8011, Halifax, NS, B3K 6R8, Canada; 2 School of Nursing and Midwifery, Brookfield Health Sciences Complex, University College Cork, Cork, Ireland; 3 Departments of Anesthesiology, Perioperative, and Pain Medicine; and Community Health Sciences, University of Alberta, Calgary, AB, Canada; 4 IWK Health, Strengthening Transitions in Care Lab, Halifax, NS, Canada; 5 Acadia Expansion Site, Cape Breton University, Wolfville, NS, Canada; 6 School of Epidemiology and Public Health, University of Ottawa, Ottawa, ON, Canada; 7 Maritime SPOR SUPPORT Unit, Halifax, NS, Canada; 8 College of Nursing and Health Sciences, Caring Futures Institute, Flinders University, Adelaide, Australia; 9 School of Health and Human Performance, Faculty of Health, Dalhousie University, Halifax, NS, Canada; 10 School of Nursing, University of British Columbia Okanagan, Kelowna, BC, Canada; 11 School of Public Health, University of Alberta, Edmonton, AB, Canada; 12 Faculty of Nursing & Department of Community Health Sciences, University of Calgary, Calgary, AB, Canada; 13 Holland Bloorview Research Institute, Holland Bloorview Kids Rehabilitation Hospital, Toronto, ON, Canada; 14 Izaak Walton Killam Health Centre, Halifax, NS, Canada; 15 Faculty of Nursing, University of Alberta, Edmonton, AB, Canada; 16 Division of Adolescent Medicine, Hospital for Sick Children, Toronto, ON, Canada

**Keywords:** realist review, implementation, sustainability, knowledge translation, youth, health care transitions

## Abstract

**Background:**

Supporting youth and their caregivers during the transition from pediatric to adult health care is a priority across Canada. Many transition in care (TiC) innovations exist, yet these innovations often fail to be effectively implemented and/or sustained. Knowledge translation (KT) interventions, such as developing educational materials and identifying champions, are used to promote the uptake of innovations into clinical practice. However, there is limited information on what, when, and how these KT interventions are used to implement and sustain TiC innovations.

**Objective:**

This paper presents the protocol for a realist review aiming to understand what KT interventions work, how they work, for whom, and under what circumstances to support the implementation and sustainability of TiC innovations for youth moving from pediatric to adult health care. The objectives are to (1) identify and map KT interventions and (2) develop initial program theories.

**Methods:**

We will follow Pawson’s 5 iterative steps for realist reviews, integrating aspects of rapid realist review methodology. We will adhere to the RAMESES (Realist and Meta-narrative Evidence Syntheses–Evolving Standards) quality standards for realist synthesis. Using an integrated KT approach, we will leverage the lived and professional expertise of a team of knowledge users and researchers.

**Results:**

This project has received funding from Canadian Institutes of Health Research, starting in January 2026. A research partner team of 24 people with lived and professional expertise in transition has been assembled, as well as a team of 18 scientific members. Step 1 of the review is underway. The review is anticipated to be completed within 12 months.

**Conclusions:**

Using a theory-driven approach, this realist review will result in initial program theories about the underlying mechanisms, contextual factors, and processes within KT interventions that influence implementation and sustainability outcomes of TiC innovations for youth and their caregivers. A subsequent explanatory mixed methods realist evaluation with a multiple comparative case study design will test and refine initial theories. This research will be important to inform future TiC innovations for diverse health contexts across Canada and beyond.

## Introduction

### Background

In Canada, more than 20% of children and youth live with at least 1 chronic health condition [1]. More than 90% of these youth will transition to adult services [2,3]. Transition from pediatric to adult care is defined as a purposeful, planned movement of youth with chronic medical conditions from child-centered to adult-oriented health care [4]. This is supported by individualized transition planning, coordinated transfer, and secure attachment to adult health services [4]. Youth and their caregivers who are not supported through this transition face poorer clinical outcomes [5-12] and increased use of acute care services [9,13]. These outcomes are exacerbated by barriers related to social and structural determinants of health that influence the transition process, such as gender, race, and ethnicity [14]. Poor transition further impacts the health system with increased use of health service resources and higher expenditures [15-17].

In response to these stark outcomes, the transition from pediatric to adult health care has been deemed a critical health system issue in Canada [18]. The transition in care (TiC) process is complex, with many interconnected behaviors and processes. As such, it requires multicomponent and multilevel innovations to address the individual, organizational, and system-level barriers and facilitators to transition. Many innovations exist for the transition from pediatric to adult care (eg, patient and peer navigation programs, transition checklists, and guidelines), and millions of dollars have been invested to support TiC [19-22]. However, these innovations often fail to be effectively implemented and sustained [18,23]. A 2020 systematic review highlighted that TiC research has focused on innovation-specific outcomes without addressing implementation and/or sustainability outcomes [23]. There is a need to understand how to enable TiC innovations to be sustained in clinical practice, to achieve long-term impact on youth, caregiver, and health system outcomes.

The implementation and sustainability of health innovations is a major challenge across health system contexts [24]. Implementation focuses on putting an innovation into use [25]. Sustainability focuses on the continuation of an innovation after a defined period, whereby adaptation of the innovation and behavior may continue to produce improved practices and maintain benefits for individuals and systems [26]. Knowledge translation (KT) interventions are strategies designed to promote the implementation and sustainability of innovations into clinical practice and policy [27]. While the term “KT interventions” is used widely in Canada, we recognize that these strategies may also be referred to as “implementation science interventions.” Common KT interventions include education, audit and feedback, and clinical champions [27]. Evidence exists on a range of KT interventions [28-31] to increase the implementation of innovations with different health care providers [32-34], for various health conditions [35,36], and across different contexts [37,38]. While comparatively less abundant, there is growing evidence on the use of KT interventions for sustaining innovations in practice [39,40]. Despite TiC research and implementation and sustainability science literature, there is limited empirical evidence on what, when, and how KT interventions are used to implement and sustain TiC innovations for youth and their caregivers [41]. Efforts are needed to address this gap and reap the long-term patient, health care provider, and health system benefits of TiC innovations. This knowledge will advance researchers’ and health system implementers’ ability to design and implement KT interventions for TiC innovations that directly target implementation and sustainability determinants and outcomes [42].

### Review Aim and Objectives

This paper presents the protocol for a realist review aiming to understand what KT interventions work, how they work, for whom, and under what circumstances to support the implementation and sustainability of TiC innovations for youth and their caregivers transitioning from pediatric to adult health care. The objectives are to

- Identify and map KT interventions: using a theory-driven approach, identify the range of KT interventions that have been used to implement and sustain TiC innovations for youth and their caregivers transitioning to adult care
- Develop initial program theories (IPTs): examine the underlying mechanisms, contextual factors, and processes within the identified KT interventions that influence implementation and sustainability outcomes of TiC innovations.

## Methods

### Methodology

Realist reviews are an interpretive, theory-driven approach to evidence synthesis that uses multiple sources of evidence, with a strong emphasis on key knowledge user involvement to provide experiential explanations of how interventions might work [43]. This approach is particularly suited to understanding complex interventions, such as KT interventions, where effectiveness depends on context and underlying mechanisms. Central to realist reviews is the development and refinement of program theories, which articulate how, why, and under what circumstances an intervention produces specific outcomes [44]. These theories are structured using the context (C), mechanism (M), and outcome (O; CMO) framework, whereby an intervention achieves an outcome because certain mechanisms are triggered in particular contexts [43]. In this review, we will identify and refine program theories for KT interventions supporting the implementation and sustainability of transition from pediatric to adult care innovations.

### Study Design

Integrating aspects of Saul et al’s [44] rapid realist review methodology, we will follow Pawson’s [45] five steps for realist reviews, which include (1) define the review scope, (2) develop IPTs, (3) evidence search, (4) selection and appraisal, and (5) data extraction and synthesis. As the aim of the realist review is to develop IPTs, these steps are iterative and can overlap. This realist review will be guided by the RAMESES (Realist and Meta-narrative Evidence Syntheses–Evolving Standards) reporting standards, which will be used throughout the review process to ensure transparency and rigor in the development, testing, and refinement of program theories. In particular, the guidelines will inform how CMO configurations are identified and reported, how iterative theory development is documented, and how synthesis decisions and analytical reasoning are made explicit across stages of the review [46,47].

### Integrated KT Approach

**Figure 1.**
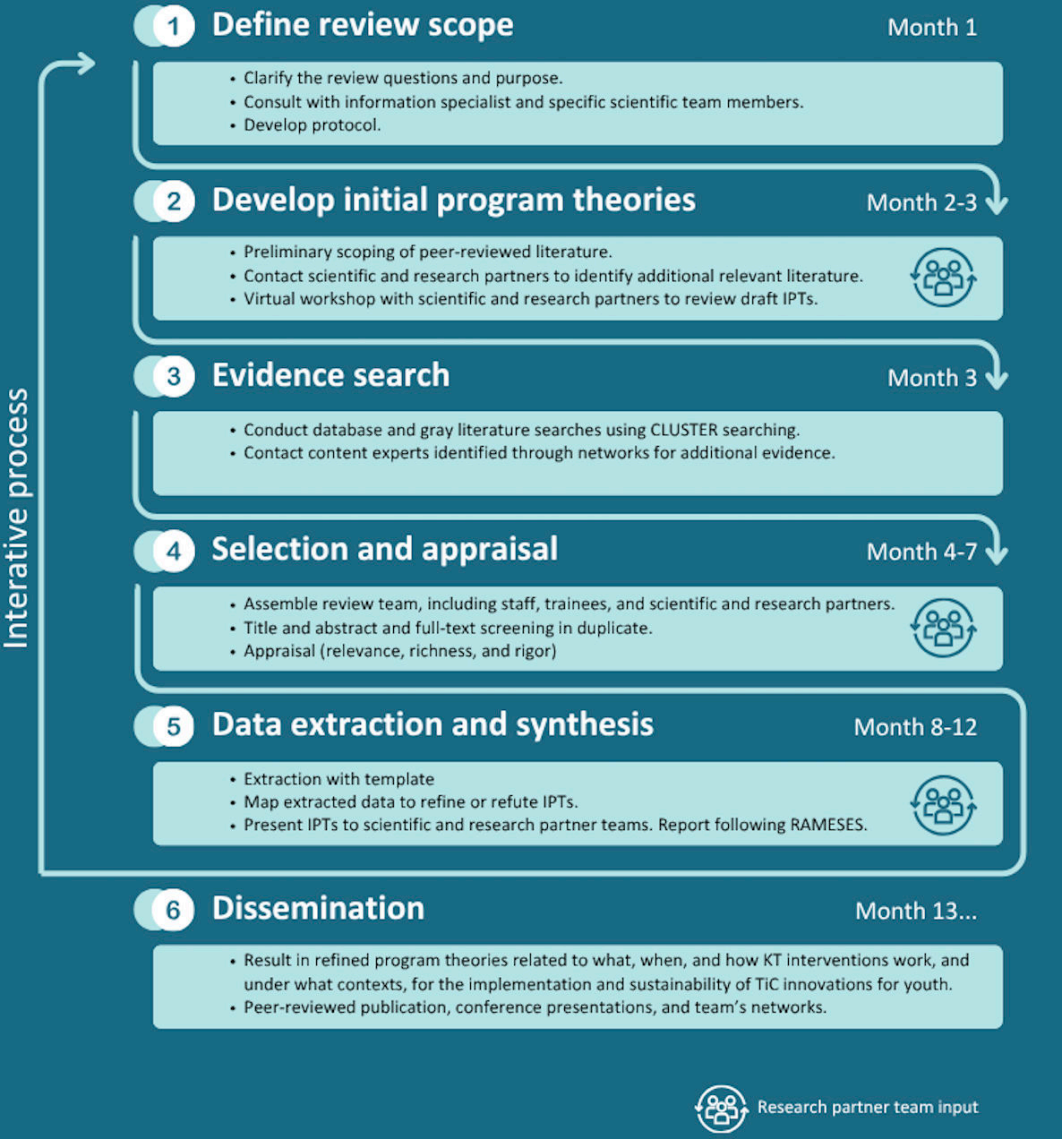
Realist review stages with integrated knowledge translation (iKT) approach. CLUSTER: Citation tracking, tracing lead authors, identifying unpublished materials, Google Scholar searching, theory tracking, ancestry searching for early examples, and follow-up of related projects; IPT: initial program theory; RAMSES: Realist and Meta-narrative Evidence Syntheses–Evolving Standards; TiC: transition in care.

An integrated knowledge translation (iKT) approach will be embedded throughout to coproduce this research with those directly involved in and affected by TiC [48]. The project’s scientific team includes researchers with expertise in realist review methodology, KT, and TiC. The research partner team consists of people with lived and professional expertise, including caregivers and youth with chronic conditions, health care providers, health system decision-makers, and community partners. Our iKT approach will be guided by the Research Quality Plus for Co-Production framework [49]. Research Quality Plus for Co-Production provides a practical approach for designing, managing, and evaluating research coproduction. Figure 1 shows the iterative stages of our review, highlighting the primary steps in which the research partner team will be engaged.

### Step 1: Define the Review Scope

The implementation and sustainability of health innovations is a major challenge for researchers and clinicians alike [24]. TiC innovations refer to evidence-based policies, practices, or programs intended to improve the transition from pediatric to adult health care, such as patient transition planning or self-management programs [50]. This review is focused on the use of KT interventions for TiC innovations. KT interventions are strategies used to facilitate the uptake and long-term maintenance of TiC innovations [27]. For example, types of KT interventions that could be examined in this review include training and educating interest-holders (eg, develop toolkits and checklists), using evaluative strategies (eg, assess barriers and facilitators), developing interest-holder interrelationships (eg, identify and prepare champions), engaging consumers (eg, patient and family member advisory groups), and using financial strategies (eg, secure grant or private funding or develop business cases) [28-38,40]. We will examine mechanisms to explain how and why specific KT interventions succeed or fail under different conditions [41].

In realist terms, mechanisms are the underlying processes, structures, or human responses that operate within particular contexts to generate outcomes. We are interested in outcomes that measure the impact of KT interventions on TiC innovation implementation and/or sustainability. We anticipate this review will examine implementation outcomes such as acceptability, adoption, implementation cost, and feasibility [51]. Sustainability outcomes, for example, could include the continued benefits of the TiC innovation for patients and staff, maintenance of partnerships, maintenance of new policies created because of the TiC innovation, continued TiC innovation activities, increased awareness of the TiC problem, and replication and scale-up of the TiC innovation [40,52]. This preliminary scope of the review has been informed by prior TiC and KT research conducted by members of the scientific team [53-57]. Defining the review scope will be an iterative process, progressively refined in response to emerging evidence and expert input to ensure the review remains focused, relevant, and actionable.

### Step 2: Develop Draft IPTs

A central component of the realist research cycle is the development, testing, and refinement of program theories, which articulate how and why an intervention is expected to work [45]. IPTs represent early hypotheses about how, for whom, why, and under what contexts complex interventions work or do not work [45,58]. Drafting IPTs is the first step in a theory-driven realist research cycle [45]. To develop these draft IPTs, we will begin with a preliminary scoping of peer-reviewed literature in MEDLINE to map the breadth and nature of existing evidence. The preliminary search strategy (Multimedia Appendix 1), developed by a health services information specialist (LB), will use key concepts related to pediatrics, TiC, and KT interventions informed by the Expert Recommendations for Implementing Change taxonomy of implementation strategies [59]. An initial purposive sample of approximately the first 200 titles and abstracts ranked by relevance will be screened to identify literature that can support IPT development. In parallel, members of the scientific and research partner teams will be invited by email to identify peer-reviewed literature [44]. This targeted and purposive approach aligns with realist synthesis methodology, which emphasizes iterative, theory-driven searching and conceptually rich and relevant evidence over exhaustive screening during early stages of theory generation [60].

All identified “seed” literature will be extracted into Microsoft Excel, including study characteristics (eg, title, author, publication date, context, and study population), KT interventions, and data relevant to CMO configurations, including evidence of how and why outcomes are generated in specific contexts. The draft IPTs will be developed through an iterative synthesis of evidence from the “seed” literature and input from scientific and research partner team members. First, findings from extracted “seed” literature will be synthesized to generate draft explanatory “if-then” statements, representing early program theories. Prioritization of the draft IPTs will be guided by the strength of supporting evidence, including the richness and relevance of CMO data, recurrence of patterns across sources, and knowledge users’ perception of relevance to practice. Where multiple competing explanations emerge, these will be retained initially and explored further rather than excluded [61,62].

Second, draft IPTs will then be presented to scientific and research partner team members at a joint virtual workshop. The workshop will be 30 minutes and will be hosted on Microsoft Teams. A consensus-building approach will be used to review, challenge, and prioritize IPTs through structured discussion. The project leads (CEC and RF) will facilitate the workshop, and disagreements will be addressed through deliberation and re-examination of the underlying evidence. Where consensus cannot be achieved, decisions will be revisited against the extracted data, and the most empirically supported and theoretically coherent IPTs will be retained. Engaging knowledge users from the scientific and research partner teams from the outset is essential, as they are either directly impacted by the research or positioned to use the findings in practice to support TiC evidence-based intervention implementation [45].

This process of developing draft IPTs will also inform decisions regarding the scope and focus of the full realist review, including the types of transition contexts and KT interventions to be included. A formal record of decisions and discussions will be maintained. The resulting IPTs will remain provisional and will provide the initial theoretical framework for the full realist review, guiding the search strategy, study selection, data extraction, and early stages of synthesis. They will continue to be refined throughout the review, with new program theories potentially emerging as additional evidence is identified and analyzed.

### Step 3: Evidence Search

We will conduct a more comprehensive search of peer-reviewed and gray literature to further refine IPTs. Consistent with realist methodology, searching will be iterative and purposive, allowing emerging program theories to guide subsequent searches to identify evidence that can support, refute, or refine these theories. We will use CLUSTER searching (Citation tracking, tracing lead authors, identifying unpublished materials, Google Scholar searching, theory tracking, ancestry searching for early examples, and follow-up of related projects), specifically designed for realist searches, as the review progresses [63]. The preliminary MEDLINE search will be adapted based on information and input gathered in step 2. A health services information specialist (LB) will translate the MEDLINE strategy to other selected electronic databases, including but not limited to Embase (Ovid) and CINAHL (EBSCO). The search strategy will be refined with the research team and peer-reviewed by a second information specialist using the Peer Review of Electronic Search Strategies (PRESS) guideline statement [64].

Gray literature will be retrieved through targeted searches in Google. Google searches will contain similar search terms to the database search strategy. Additionally, the team will conduct hand searching of organizational websites concerned with TiC innovations for youth (eg, Children’s Healthcare Canada, Transition Hub, Got Transition, and CanChild). An existing environmental scan conducted by Children’s Healthcare Canada of TiC innovations in Canada will be screened to identify any use of KT interventions for implementation and/or sustainability [53]. We will seek other evidence by contacting additional content experts identified through scientific and research partner team members’ networks. This step is critical to propel the search process while ensuring that evidence that is relevant to theory building is not overlooked [44]. The reference lists of all included documents will be hand searched to identify additional relevant citations for inclusion. Further searches will be undertaken, and inclusion criteria and search terms will be adjusted as new concepts emerge. Evidence searches will stop when additional evidence no longer meaningfully contributes insights to program theory development, refinement, and testing.

### Step 4: Selection and Appraisal

The review team will comprise researchers, postdoctoral researchers, and master’s- and doctoral-level trainees with experience in health-related research and implementation science. We will invite the scientific and research partner team members to participate in screening if they are interested. Following the evidence search, all identified records retrieved from the electronic database search will be uploaded into Covidence (Veritas Health Information) and duplicates removed.

Two independent reviewers will screen titles and abstracts. Records deemed eligible for full-text review will be assessed by 2 independent reviewers. Gray literature screening will be managed in Microsoft Excel. Google searches will be executed by a group of independent reviewers who will input potentially relevant results into Excel. Google search results will be screened until the reviewer advances 2 pages past the last potentially relevant result clicked. All potentially relevant gray literature will be screened against inclusion criteria by 2 independent reviewers. Reasons for exclusion will be reported. Any conflicts between reviewers at each stage of the study selection process will be resolved by a third reviewer or through team consensus. The review team will meet regularly to ensure a common understanding and to identify any iterative changes to the inclusion criteria. A flow diagram, adapted from the PRISMA-ScR (Preferred Reporting Items for Systematic Reviews and Meta-Analyses Extension for Scoping Reviews), will show the identification, screening, and inclusion of documents for the review [65].

Peer-reviewed and gray literature that describes the use of KT interventions for the implementation and/or sustainability of TiC innovations will be eligible for inclusion. TiC innovations must be within a health care context and target youth (aged 15‐24 years) diagnosed with one or more chronic conditions who have already or will transfer from pediatric to adult health care. This age range aligns with the definition of “youth” under the United Nations and recommendations for caregivers and health care providers to begin the transition process as early as age 14 [66]. Literature published in any language will be included where translation to English is possible using artificial intelligence translation software DeepL Translator. Inclusion criteria are detailed in Table 1. These a priori criteria may be narrowed further once the extent and nature of the literature is determined in steps 1 and 2.

**Table 1.** Inclusion criteria.

	Inclusion criteria
Population	Youth (aged 15‐24 years) diagnosed with one or more chronic conditions (physical, mental, or developmental) who have been or will transfer from pediatric to adult health care Caregivers, including parents or partners, of patients who have been or will transfer from pediatric to adult health care Multidisciplinary health care providers involved in supporting the transition from pediatric to adult health care
Context	Any health care context, including pediatric and adult, inpatient, ambulatory, or primary care Any geographic location
Concept	Describes the use of knowledge translation interventions for the implementation and/or sustainability of transitions in care innovations
Study type	All study types No restriction on publication date, geographic location, or language

Realist reviews require a series of critical judgments of relevance, richness, and rigor to assess the fit of documents in addressing the review questions [44-47]. This appraisal process ensures that, where a high volume of documents are eligible at title and abstract screening, priority is given to those that provide sufficient detail to identify and develop demi-regularities, meaning recurring patterns in CMO configurations [67,68]. In this way, included documents are those that most directly advance understanding of how and why outcomes are generated across different contexts. A full-text screening tool will be developed, informed by Dada et al’s [69] considerations for relevance, richness, and rigor appraisal.

Relevance determines whether a document is relevant to the topic area or program theory. Relevance will be assessed based on whether the document contains data relevant to implementing and/or sustaining TiC innovation using an identified KT intervention [69]. Documents that examine concepts closely related to KT interventions but do not link them to TiC innovation implementation outcomes, as outlined by Proctor et al [51] (eg, acceptability, adoption, cost, and feasibility), or sustainability outcomes, as outlined by Lennox et al [52] (eg, continued benefits, maintenance of partnerships and policies, increased awareness, and replication and scale-up), will be excluded. Inclusion and exclusion criteria for relevance will be outlined in the screening tool.

Recently, richness has been suggested as an important category of appraisal for realist reviews related to complex interventions [63]. Richness refers to the degree of theoretical and conceptual development that explains how an intervention is expected to work [63,69]. Richness will be assessed using Waldron et al’s [70] criteria and scoring system to determine whether the document can meaningfully contribute to theory development, refinement, or testing. Documents that provide limited to no evidence of interest will be scored low (levels 0‐1), whereas documents that provide sufficient detail about how the KT intervention used was expected to work will be scored high (levels 2‐4).

Rigor is important to ensure the method used to develop the evidence is trustworthy and the program theory is coherent [69,71]. Unlike systematic reviews, the RAMESES does not recommend using formal checklists to assess rigor [46,47]. We will assess rigor at the level of the relevant data and program theory described in the record, not of the study or intervention as a whole. We will outline guiding questions, such as those described by Dada et al [69] and Morton et al [72] for reviewers to consider in determining the level of rigor as high, medium, or low. We will consider evidence of lower quality where relevance and richness for program theory development are assessed as high.

### Step 5: Data Extraction and Synthesis

The research team will determine that the review has reached its conclusion when the evidence retrieved no longer adds new knowledge to the understanding of how KT interventions work or do not work for the implementation and sustainability of TiC innovations. This is in keeping with descriptions of saturation in realist reviews described in the literature [44].

Data will be extracted from documents included in the review using a data extraction template in Covidence. The template will provide a standardized approach to ensure that information relevant to the core areas of the IPTs is thoroughly extracted across all documents. Scientific and research partner team members will critique the extraction template before the extraction process begins, and modifications will be made based on consensus. The template will include fields such as study characteristics (eg, study design, year of publication, and geographic location); components of the TiC innovation; equity or cultural considerations; sex, gender, and other identifying characteristics of study participants; components of the KT interventions; theoretical or conceptual frameworks; reported barriers and enablers to implementation and/or sustainability; implementation and/or sustainability outcomes; and interactions between contexts and mechanisms [44]. Pilot extractions will be conducted with all members of the review team. All members will extract the same 5 documents [44]. Members will meet to compare their extractions for consistency and discuss any necessary adjustments to the template. Further extractions will be conducted by 1 individual team member and verified by another independent team member. Any conflicts between extractors will be resolved by a third team member or through team consensus.

Synthesis of evidence will be guided by realist logic to answer the review questions. The extracted data will be mapped to identify areas of confirmation, conflict, or new information that is not addressed by the draft IPTs. Findings will be reported narratively using the CMO heuristic to explain causal links between contexts, mechanisms, and outcomes. Following data synthesis, tested and refined program theories and CMO configurations will be reviewed by the scientific and research partner team to ensure they represent their lived and professional experience. The results will be reported according to the RAMESES reporting standards [46,47].

### Ethical Considerations

Ethics approval is not required for this realist review as it is secondary research that will synthesize evidence from primary studies and gray literature.

## Results

This project is supported by a Canadian Institutes of Health Research Project Grant received in January 2026. The review is anticipated to be completed within 12 months. The initial stage of defining the review scope is currently underway. We have assembled a team of 18 scientific team members. We have recruited and onboarded 24 research partner team members, including youth patients, caregivers, health care providers, health system decision-makers, TiC innovation developers, and community partners. The review will result in program theories on what, when, how, and under what contexts KT interventions support the implementation and/or sustainability of TiC innovations for youth. We will disseminate the findings through an open-access peer-reviewed publication, conference presentations, our international and national networks in KT and TiC, and other avenues to be identified by the research partner team.

## Discussion

Many TiC innovations have been developed to support the transition from pediatric to adult health care; however, these innovations often fail to be implemented and/or sustained [41,42]. Using a theory-driven approach, this realist review will identify the range of KT interventions that have been used to implement and sustain TiC innovations. The review will examine the underlying mechanisms, contextual factors, and processes within KT interventions that could influence implementation and sustainability outcomes of TiC innovations. While the focus of this review is on youth health, the resulting theories may be relevant to other TiC contexts and could be tested in different contexts and populations.

Unlike other types of evidence synthesis, such as systematic reviews, which use comprehensive search methods to assess the effectiveness of interventions, realist reviews do not attempt to identify all relevant literature or to determine whether interventions work or do not work [45]. Realist reviews follow a purposeful trail of evidence to unpack the “black box” between underlying mechanisms and generated outcomes for complex health system interventions across different contexts. As such, realist synthesis aims to produce transferable rather than generalizable findings [45]. This means findings may not apply across all settings, and readers must judge the fit between the synthesized theory and their local context. To support the transferability of the findings, our approach is fundamentally rooted in Pawson’s realist synthesis methodology but adopts aspects of rapid realist reviews for efficient evidence generation [44]. Furthermore, a key strength of this review is the emphasis on knowledge user involvement through the integration of the research partner team. Drawing on the expertise of this team will ensure the identification of relevant peer-reviewed and gray literature on TiC innovations and provide experiential explanations of how KT interventions might work. We will leverage the realist review and KT expertise of the scientific team. We recognize that mobilizing large scientific and research partner teams may lead to challenges with sustained engagement and potential power imbalances among team members. To mitigate this, the research partner team will be co-led by a youth patient partner and a terms of reference document will be co-developed at the outset to guide the partnership and trust-building among the team.

The results of this realist review will directly inform subsequent phases of the project. We will conduct an explanatory mixed methods realist evaluation with a multiple comparative case study design to test IPTs produced from the realist review. Three real-world TiC innovations targeting youth patients will function as independent “cases” bounded by geographic location (3 Canadian provinces), patient population, and time from initial implementation. The project will culminate by co-developing recommendations for how to implement and sustain TiC innovations in practice. These recommendations will be valuable for researchers and health system implementers to design and implement KT interventions that directly support implementation and sustainability of TiC innovations. This research will be critical to inform future youth TiC innovations for a range of health conditions across Canada and internationally, ensuring long-term benefits to patients, health care providers, and health system outcomes.

## Supplementary material

10.2196/99731 Multimedia Appendix 1 MEDLINE search strategy.

10.2196/99731 Checklist 1 RAMESES II reporting standards for realist evaluations.

10.2196/99731 Peer Review Report 1 Peer review report by the Knowledge Translation Research Review Committee, Canadian Institutes of Health Research (CIHR).

## Abbreviations:

CMO: context, mechanism, and outcome
iKT: integrated knowledge translation
IPT: initial program theory
KT: knowledge translation
PRESS: Peer Review of Electronic Search Strategies
PRISMA-ScR: Preferred Reporting Items for Systematic Reviews and Meta-Analyses Extension for Scoping Reviews
RAMESES: Realist and Meta-narrative Evidence Syntheses–Evolving Standards
TiC: transition in care
