# Supplementary material for: Knowledge Translation Interventions for Youth Health Care Transitions: Protocol for a Realist Review
Source: JMIR Res Protoc. 2026 Jul 15;15:e99731. doi: 10.2196/99731 (PMC13372076; doi:10.2196/99731)
Supplement: Multimedia Appendix 1 [file resprot-v15-e99731-s001.docx]

MEDLINE

April 29^th^ 2026

| 1 | exp Child/ or exp Pediatrics/ or (child* or kid or kids or girl or girls or boy or boys or juvenile* or minors or p?ediatric?).ti,ab,kf. | 3305078 |
| --- | --- | --- |
| 2 | Adolescent/ or (teen* or youth* or adolescen* or young adult*).ti,ab,kf. | 2637347 |
| 3 | or/1-2 | 4625766 |
| 4 | Transition to Adult Care/ | 2746 |
| 5 | (transition* adj2 care).ti. | 3703 |
| 6 | (transition* adj2 healthcare).ti. | 234 |
| 7 | (transition* adj2 p?ediatric care).ti. | 14 |
| 8 | (transition* adj2 youth care).ti. | 0 |
| 9 | (transition* adj2 adult care).ti. | 243 |
| 10 | or/4-9 | 6217 |
| 11 | implement*.ti. | 99915 |
| 12 | (program* or service* or support*).ti. | 651017 |
| 13 | (strateg* or approach*).ti. | 700849 |
| 14 | evaluat*.ti. | 773422 |
| 15 | (knowledge translation or kt or ikt).ti. | 2175 |
| 16 | knowledge mobili*ation.ti. | 136 |
| 17 | or/11-16 | 2131819 |
| 18 | 3 and 10 and 17 | 714 |
