# Supplementary material for: Knowledge Translation Interventions for Youth Health Care Transitions: Protocol for a Realist Review
Source: JMIR Res Protoc. 2026 Jul 15;15:e99731. doi: 10.2196/99731 (PMC13372076; doi:10.2196/99731)
Supplement: Peer Review Report 1 [file resprot-v15-e99731-s003.pdf]

**Review Type / Type d'évaluation:**	Reviewer 1 / Évaluateur 1
**Name of Applicant / Nom du chercheur:**	Cassidy, Christine
**Application No. / Numéro de demande:**	526945
**Agency / Agence:**	CIHR/IRSC
**Competition / Concours:**	Project Grant/Subvention Projet
**Committee / Comité:**	Knowledge Translation Research/Recherche sur l'application des connaissances
**Title / Titre:**	I-STiCK: Implementing and Sustaining Transition in Care Innovations for Youth using Knowledge Translation Interventions

Adjudication Criteria/Critères de sélection

Initial Score/Cote Initiale: 4.5

Top/Bottom Selection/Groupe supérieur/inférieur

- (●) **Top/Groupe supérieur**
- ( ) **Bottom/Groupe inférieur**

**Review Type / Type d'évaluation:**	Reviewer 1 / Évaluateur 1
**Name of Applicant / Nom du chercheur:**	Cassidy, Christine
**Application No. / Numéro de demande:**	526945
**Agency / Agence:**	CIHR/IRSC
**Competition / Concours:**	Project Grant/Subvention Projet
**Committee / Comité:**	Knowledge Translation Research/Recherche sur l'application des connaissances
**Title / Titre:**	I-STiCK: Implementing and Sustaining Transition in Care Innovations for Youth using Knowledge Translation Interventions

Summary of Application/Résumé de la demande:

I-STiCK: Implementing and Sustaining Transition in Care Innovations for Youth using Knowledge Translation Interventions. Transition in HC for those leaving child/youth services is complex with challenges due to the complexity of healthcare and social systems – which impact LTO, sustainability of TiC is fraught with challenges. With so many transition guides, pathways etc. globally the question focuses on examining what-when- and how KT interventions work (or not) and what are the circumstances/factors/contexts for implementation and sustainability. This work involves developing program theory [MM Realist Evaluation] – testing these with 3 TiC innovations (within current programs under evaluation) and refining theories and co-developing [explanatory multiple case study research design; QR interviews and DA of tracking forms], and recommendations for implementation and sustainability TiC in Canada [knowledge exchange]. TiC is happening across Canada, had huge financial investment – TiC includes a wide range of approaches, has variable success and limited sustainability, which risks young people (with chronic conditions) achieving their full potential – likely limitations in accessing what they need in ways that support wellness and future well-being as they become adults. Against this backdrop the organization, delivery, and evaluation of TiC principles that support effectiveness and promote sustainability are unclear – hence this study examines what works (and why – replicability etc.). Noted that the questions from previous reviews were answered clearly which have strengthened the application.

**Review Type / Type d'évaluation:**	Reviewer 1 / Évaluateur 1
**Name of Applicant / Nom du chercheur:**	Cassidy, Christine
**Application No. / Numéro de demande:**	526945
**Agency / Agence:**	CIHR/IRSC
**Competition / Concours:**	Project Grant/Subvention Projet
**Committee / Comité:**	Knowledge Translation Research/Recherche sur l'application des connaissances
**Title / Titre:**	I-STiCK: Implementing and Sustaining Transition in Care Innovations for Youth using Knowledge Translation Interventions

Strengths and Weaknesses/Forces et faiblesses:

Strengths

International team and across Canada, very experienced members within the group.

Evidence of prior work and career trajectory and development in this area clear. Wide range of collaborators who have, by note of their more recent publications, continued and sustained work in this area. Evidence of expertise in methodological approaches noted applied to transition – KT interventions for implementation and sustainability, research co-production, realist evaluation. Therefore, clear evidence of ability and outcomes that can be shared with Canadian and international audiences.

Timely grant if to receive funding to progress work and outputs.

There is a very honest position that the fragmentation in TiC has resulted in the splintering of approaches which if not addressed will result in transition continuing to fail young people/adults. The need for diverse groups/team/providers/KU etc. to work in collaborations vs. competitive or 'non' networked teams is a real threat to work in this area. I applaud the team for sharing this narrative – with this rich team there is an opportunity to turn the tide and demonstrate how to do such work as well as achieve the TiC outcome of pinpointing principles of meaningful sustainability that stand the test of time.

Objective one approach is clear and evidenced.

Objective two – focuses on the ways in which the three existing programs and application of the theory models can be tested – with a diverse group of caregivers/youth/providers and health system leaders.

Objective three – is well laid out with evidence of approach in the application.

Co-production, co-researchers etc. links to the SPOR principles and education to be offered. The emotional impact and emotional contribution of this work can be significant and there is an impact on the individuals (lay and professional), how is this being navigated and supported?

KT outputs are inclusive and draw in the existing networks and groups – which many team members are connected with, which is evidence of the position of drawing together a functional and productive network.

In person and use of virtual noted along with acknowledging the large team and impact on its own engagement and sustainability processes.

The study has the capacity to have an impact in this area.

Opportunities for consideration/ weakness

Team communication – sound and interesting to understand how this will work with such a big group. Consensus is the base for decision making – articulating the specifics of this could be helpful as it can be itself a complex concept to unpack in real terms.

IKT panel (how many 3.0) have shared educational and learning opportunity, how does this reflect equity and inclusion for those that are limited by complex social factors etc. Co-production (while noted as a model that has evidence of working in the area) with the IKT panel will focus on the CMO hypothesis, will the shared training be sufficient to have shared understanding and language to do this work as co-production – even with NGT (how will this be 'checked' – so that those on the IKT are not left behind and remain engaged and able to contribute).

The IKT panel youth co-lead/caregiver partner has a huge task, is this the 'professional patient/caregiver' in such a position

and what unique supports are in place for this person(s). Just a question how will diversity show up here?

**Review Type / Type d'évaluation:**	Reviewer 1 / Évaluateur 1
**Name of Applicant / Nom du chercheur:**	Cassidy, Christine
**Application No. / Numéro de demande:**	526945
**Agency / Agence:**	CIHR/IRSC
**Competition / Concours:**	Project Grant/Subvention Projet
**Committee / Comité:**	Knowledge Translation Research/Recherche sur l'application des connaissances
**Title / Titre:**	I-STiCK: Implementing and Sustaining Transition in Care Innovations for Youth using Knowledge Translation Interventions

Budget Recommendation/Recommandation budgétaire:

no concerns

Please indicate your appraisal of the integration of sex as a biological variable as a strength, weakness, or not applicable to the proposal./Prière de sélectionner une option pour donner votre évaluation de l'intégration du sexe comme variable biologique en tant que point fort ou point faible de la proposition, ou en tant qu'élément non applicable à la proposition.

- ◉ **Strength/Point fort**
- ○ **Weakness/Point faible**
- ○ **Not applicable/Non applicable**

Please indicate your appraisal of the integration of gender as a socio-cultural determinant of health as a strength, weakness, or not applicable to the proposal./Prière de sélectionner une option pour donner votre évaluation de l'intégration du genre comme déterminant socioculturel de la santé en tant que point fort ou point faible de la proposition, ou en tant qu'élément non applicable à la proposition.

- ◉ **Strength/Point fort**
- ○ **Weakness/Point faible**
- ○ **Not applicable/Non applicable**

Sex and/or Gender Considerations/Notions de sexe et/ou de genre:

The group considers GBA+ noting examples of specific consideration team structure (17 CVs attached) – considering privilege and IKT panel recruitment, trainees and KU. Interviews – diverse sample to max. variation across social and identity, linked to NS context (approach to recruitment as an inclusive exercise not provided but note word restrictions), Data analysis – noted examples using software to explore SSF and Knowledge exchange – co-design process grounded in key principles—support from SPOR and SAGER guidelines.

Privilege and position noted (pg 9). While the inclusion considers drawing for underrepresented groups – how does the team plan to embed gender-transformative approaches that address the root causes of gender-based health inequities, promoting fairness and improving health outcomes that are part of TiC similar to other HC practices. How will invitation to IKT be inclusive, SPOR education adapted to be suitable for a wider audience with a wide range of skills and accessibility complexities? How will gendered and age relations be managed with the study in establishing, managing and growing (and eventually closing or adapting) the IKT panel?

**Review Type / Type d'évaluation:**	Reviewer 2 / Évaluateur 2
**Name of Applicant / Nom du chercheur:**	Cassidy, Christine
**Application No. / Numéro de demande:**	526945
**Agency / Agence:**	CIHR/IRSC
**Competition / Concours:**	Project Grant/Subvention Projet
**Committee / Comité:**	Knowledge Translation Research/Recherche sur l'application des connaissances
**Title / Titre:**	I-STiCK: Implementing and Sustaining Transition in Care Innovations for Youth using Knowledge Translation Interventions

Adjudication Criteria/Critères de sélection

Initial Score/Cote Initiale: 4.3

Top/Bottom Selection/Groupe supérieur/inférieur

- (●) **Top/Groupe supérieur**
- ( ) **Bottom/Groupe inférieur**

**Review Type / Type d'évaluation:**	Reviewer 2 / Évaluateur 2
**Name of Applicant / Nom du chercheur:**	Cassidy, Christine
**Application No. / Numéro de demande:**	526945
**Agency / Agence:**	CIHR/IRSC
**Competition / Concours:**	Project Grant/Subvention Projet
**Committee / Comité:**	Knowledge Translation Research/Recherche sur l'application des connaissances
**Title / Titre:**	I-STiCK: Implementing and Sustaining Transition in Care Innovations for Youth using Knowledge Translation Interventions

Summary of Application/Résumé de la demande:

Importance and Impact: The Implementing and Sustaining Transition in Care Innovations for Youth using Knowledge Translation Interventions (I-STiCK) project aims to examine what, when, and how KT interventions work or do not work, and under what contexts for the implementation and sustainability of transition in care (TiC) innovations for youth transitioning from pediatric to adult care. The objectives of the project are threefold: 1) to develop initial program theories on what, how and under which contexts KT interventions work or not to support the sustainability of TiC innovations for youth; 2) to test these initial program theories in the context of three TiC innovations in Alberta, Ontario, and Nova Scotia; 3) to refine these initial program theories and co-develop recommendations for the implementation and sustainability of TiC innovations for child and youth health in Canada.The proposed project builds upon previous works from the investigators related to the development and implementation of innovative interventions to support child and youth care, as well as transition to adult care.

Methods: The proposed project is based on the RQ+ Co-Pro Framework, a co-production approach, a model of collaborative research that responds to knowledge use needs, in line with the integrated knowledge translation (iKT) approach. The proposed methodology is a mixed-methods realist evaluation comprising a realist knowledge synthesis, a multiple comparative case study, and a 2 half-days co-design in-person workshop gathering 20 participants. Three cases, consisting of TiC innovations from three provinces (Nova Scotia, Ontario, Alberta) will be analysed through a multiple comparative case study design. An iKT panel, comprising youth, caregivers, pediatric and adult care providers, health system administrators, and community partners, will be involved at every stage of the research. The iKT panel will notably contribute to refine the Context-Mechanisms-Outcome configurations derived from the realist review and multiple case study.

Expertise and resources: The NPA (Cassidy) is an early career researcher with expertise in implementation science and transitions in care for child and youth health. Over the past five years, the team members have built a collaborative research programme around common interests. They have an established track record of working with patients, families, healthcare providers, and health system leaders to co-design, implement, evaluate, and sustain evidence-based innovations. Team members are from various Canadian and international institutions and bring complementary expertise in transitions from youth to adult care, realist review and realist evaluation methods, implementation science, codesign and research co-production. Knowledge user partners include youth, caregivers and health system leaders.

The Strengthening Transitions in Care Research Lab in the IWK's Children's Health Building will act as the main project hub with administrative staff, trainees, equipment (videoconferencing and analysis software) and space (offices and meeting rooms) to support all grant activities. To support the interprovincial and international collaborative research, the team will use MS Teams functionalities to support chat-based collaboration with document sharing, virtual meetings, and project management applications. The project will be supported by a research manager, who will oversee study progress, timelines, manage administrative tasks and team communication, to ensure timely completion of deliverables. The Co-PAs will co-supervise the research staff and meet weekly to review deliverable progress and identify risks and mitigation strategies.

**Review Type / Type d'évaluation:**	Reviewer 2 / Évaluateur 2
**Name of Applicant / Nom du chercheur:**	Cassidy, Christine
**Application No. / Numéro de demande:**	526945
**Agency / Agence:**	CIHR/IRSC
**Competition / Concours:**	Project Grant/Subvention Projet
**Committee / Comité:**	Knowledge Translation Research/Recherche sur l'application des connaissances
**Title / Titre:**	I-STiCK: Implementing and Sustaining Transition in Care Innovations for Youth using Knowledge Translation Interventions

Strengths and Weaknesses/Forces et faiblesses:

Main strengths: The project has a strong conceptual anchoring and could contribute to empirical and methodological developments in implementation science. Conducting realist evaluations requires time and specialized resources and this team is very well equipped to succeed in this regard. It is also interesting to consider implementation and sustainability on a continuum and explore what factors influence the integration of innovations into routine care and in which context. The comparative multiple case study research would be very informative to compare implementation and sustainability conditions across contexts. The findings from this multicenter realist evaluation would be informative not only for the TiC domain, but also for many other health care innovations.

Main weaknesses: The proposed realist review would include both peer-reviewed and grey literature, which could lead to an impressive amount of data sources. Date of publication, country of implementation and language of publication are not mentioned in the inclusion/exclusion criteria. It could be relevant to set limits regarding the countries or publication date for instance, to avoid a large and heterogeneous pool of potentially eligible publications.

**Review Type / Type d'évaluation:**	Reviewer 2 / Évaluateur 2
**Name of Applicant / Nom du chercheur:**	Cassidy, Christine
**Application No. / Numéro de demande:**	526945
**Agency / Agence:**	CIHR/IRSC
**Competition / Concours:**	Project Grant/Subvention Projet
**Committee / Comité:**	Knowledge Translation Research/Recherche sur l'application des connaissances
**Title / Titre:**	I-STiCK: Implementing and Sustaining Transition in Care Innovations for Youth using Knowledge Translation Interventions

Budget Recommendation/Recommandation budgétaire:

The budget seems appropriate for the planned activities.

**Review Type / Type d'évaluation:**	Reviewer 2 / Évaluateur 2
**Name of Applicant / Nom du chercheur:**	Cassidy, Christine
**Application No. / Numéro de demande:**	526945
**Agency / Agence:**	CIHR/IRSC
**Competition / Concours:**	Project Grant/Subvention Projet
**Committee / Comité:**	Knowledge Translation Research/Recherche sur l'application des connaissances
**Title / Titre:**	I-STiCK: Implementing and Sustaining Transition in Care Innovations for Youth using Knowledge Translation Interventions

Please indicate your appraisal of the integration of sex as a biological variable as a strength, weakness, or not applicable to the proposal./Prière de sélectionner une option pour donner votre évaluation de l'intégration du sexe comme variable biologique en tant que point fort ou point faible de la proposition, ou en tant qu'élément non applicable à la proposition.

- ○ **Strength/Point fort**
- ○ **Weakness/Point faible**
- ◉ **Not applicable/Non applicable**

Please indicate your appraisal of the integration of gender as a socio-cultural determinant of health as a strength, weakness, or not applicable to the proposal./Prière de sélectionner une option pour donner votre évaluation de l'intégration du genre comme déterminant socioculturel de la santé en tant que point fort ou point faible de la proposition, ou en tant qu'élément non applicable à la proposition.

- ◉ **Strength/Point fort**
- ○ **Weakness/Point faible**
- ○ **Not applicable/Non applicable**

**Review Type / Type d'évaluation:**	Reviewer 2 / Évaluateur 2
**Name of Applicant / Nom du chercheur:**	Cassidy, Christine
**Application No. / Numéro de demande:**	526945
**Agency / Agence:**	CIHR/IRSC
**Competition / Concours:**	Project Grant/Subvention Projet
**Committee / Comité:**	Knowledge Translation Research/Recherche sur l'application des connaissances
**Title / Titre:**	I-STiCK: Implementing and Sustaining Transition in Care Innovations for Youth using Knowledge Translation Interventions

Sex and/or Gender Considerations/Notions de sexe et/ou de genre:

The proposal adopts a gender-based approach-plus (GBA+) in its different sections. The team will integrate a GBA+ approach to sampling for maximum variation across PROGRESS-Plus variables. They will invest efforts to engage a diverse representation of co-design participants to generate equitable recommendations. They also partner with the Maritime SPOR SUPPORT Unit that will help with recruitment efforts, and their Equity, Diversity and Inclusion (EDI) Science Lead will provide guidance to ensure the co-design event is guided by leading practices in accessibility.

**Review Type / Type d'évaluation:**	Reviewer 3 / Évaluateur 3
**Name of Applicant / Nom du chercheur:**	Cassidy, Christine
**Application No. / Numéro de demande:**	526945
**Agency / Agence:**	CIHR/IRSC
**Competition / Concours:**	Project Grant/Subvention Projet
**Committee / Comité:**	Knowledge Translation Research/Recherche sur l'application des connaissances
**Title / Titre:**	I-STiCK: Implementing and Sustaining Transition in Care Innovations for Youth using Knowledge Translation Interventions

Adjudication Criteria/Critères de sélection

Initial Score/Cote Initiale: 4.1

Top/Bottom Selection/Groupe supérieur/inférieur

- (●) **Top/Groupe supérieur**
- ( ) **Bottom/Groupe inférieur**

**Review Type / Type d'évaluation:**	Reviewer 3 / Évaluateur 3
**Name of Applicant / Nom du chercheur:**	Cassidy, Christine
**Application No. / Numéro de demande:**	526945
**Agency / Agence:**	CIHR/IRSC
**Competition / Concours:**	Project Grant/Subvention Projet
**Committee / Comité:**	Knowledge Translation Research/Recherche sur l'application des connaissances
**Title / Titre:**	I-STiCK: Implementing and Sustaining Transition in Care Innovations for Youth using Knowledge Translation Interventions

Summary of Application/Résumé de la demande:

The aim of this application is to gain a better understanding of how knowledge mobilization interventions are effective in integrating innovations supporting transitions in care (ICT) for paediatric clients. The application incorporates a realist review to create an initial theory, which will then be tested with three ICT innovations in three different contexts (Alberta, Ontario and Nova Scotia). Applicants will then collaborate to develop recommendations for implementing and sustaining TIC innovations for pediatric clients.

**Review Type / Type d'évaluation:**	Reviewer 3 / Évaluateur 3
**Name of Applicant / Nom du chercheur:**	Cassidy, Christine
**Application No. / Numéro de demande:**	526945
**Agency / Agence:**	CIHR/IRSC
**Competition / Concours:**	Project Grant/Subvention Projet
**Committee / Comité:**	Knowledge Translation Research/Recherche sur l'application des connaissances
**Title / Titre:**	I-STiCK: Implementing and Sustaining Transition in Care Innovations for Youth using Knowledge Translation Interventions

Strengths and Weaknesses/Forces et faiblesses:

1) Importance and impact of research

+ NEARLY ONE IN FIVE CANADIAN CHILDREN WILL MAKE THE TRANSITION FROM PEDIATRIC TO ADULT HEALTH CARE. The candidates made an interesting demonstration of the magnitude of the issue at hand, pointing out that 20% of young people live with a chronic condition and that 18% will have to make the transition to adult services. They explain the stakes involved in a poorly executed transition, without however underlining the proportion of individuals for whom this happens (e.g. do 5% fail to make the transition?). They highlight the sums and initiatives put forward to respond to this issue, while saying that TIC tools are not implemented - it's not clear at what level.

+Les auteurs illustrent bien la complexité de la transition et le nombre des parties prenantes impliquées. Cela est déterminant dans la justification de l'utilisation de l'approche réaliste à venir.

+Les auteurs mettent l'accent sur l'importance d'une théorie de programme et son importance pour l'implantation réussie de TIC tool, ce qui est cohérent avec les avancées actuelles en science de l'implantation.

+

–

2) Approaches and methods

+The co-production methods seem solid and well organized. In particular, the explanation of power sharing and the training and involvement of ICT panel members is a strength of this application

- I am not sure if sustainability is the real outcome of the CMO, nor how sustainability is defined. Sustainability is a paradox in evidence-based practice and to me represents the continuation of implementation, so it might be useful to have a joint reflection on the real expected outcome and its definition.

- The method and goal of the environmental scan is poorly described, so it is difficult to see how it would or could inform the implementation of the TIC innovation.

- The creation of the initial theory is usually more complex than what is reflected in the proposal and involves significant discussion and work. It would have been reassuring to see this explained more in deep.

-Step 2: While it is always interesting to discuss with the end users of an innovation, in this case I am not sure that the pediatric population and their caregivers can contribute to the effectiveness of implementation strategies (but they can say whether the TIC work for them or not). Please clarify the population of interest or explain or the patient can discuss the implementation strategy.

-Did the authors consider to use the CFIR 2.0?.

+The theoretical models used are well chosen and fit together, increasing the coherence of the results.

- Since the TIC should have been implemented by 2024, it may be difficult to accurately complete the KT intervention log retrospectively. The planned and actual implementation strategy may differ, and there is no way to verify the quality of some aspects after implementation. While the structural aspect of the implementation can be documented, the lack of some very "qualitative" information can limit the accuracy of the mechanism documentation.

+ The knowledge mobilization plan presents relevant objectives and participants and is very realistic.

3) Expertise, Experience and Resources

**Review Type / Type d'évaluation:**	Reviewer 3 / Évaluateur 3
**Name of Applicant / Nom du chercheur:**	Cassidy, Christine
**Application No. / Numéro de demande:**	526945
**Agency / Agence:**	CIHR/IRSC
**Competition / Concours:**	Project Grant/Subvention Projet
**Committee / Comité:**	Knowledge Translation Research/Recherche sur l'application des connaissances
**Title / Titre:**	I-STiCK: Implementing and Sustaining Transition in Care Innovations for Youth using Knowledge Translation Interventions

+The team demonstrate a strong experience in iKT with a variety of public. The menthorship put forward interesting and reinforce the sustainability of the team.

**Review Type / Type d'évaluation:**	Reviewer 3 / Évaluateur 3
**Name of Applicant / Nom du chercheur:**	Cassidy, Christine
**Application No. / Numéro de demande:**	526945
**Agency / Agence:**	CIHR/IRSC
**Competition / Concours:**	Project Grant/Subvention Projet
**Committee / Comité:**	Knowledge Translation Research/Recherche sur l'application des connaissances
**Title / Titre:**	I-STiCK: Implementing and Sustaining Transition in Care Innovations for Youth using Knowledge Translation Interventions

Budget Recommendation/Recommandation budgétaire:

No comments

**Review Type / Type d'évaluation:**	Reviewer 3 / Évaluateur 3
**Name of Applicant / Nom du chercheur:**	Cassidy, Christine
**Application No. / Numéro de demande:**	526945
**Agency / Agence:**	CIHR/IRSC
**Competition / Concours:**	Project Grant/Subvention Projet
**Committee / Comité:**	Knowledge Translation Research/Recherche sur l'application des connaissances
**Title / Titre:**	I-STiCK: Implementing and Sustaining Transition in Care Innovations for Youth using Knowledge Translation Interventions

Please indicate your appraisal of the integration of sex as a biological variable as a strength, weakness, or not applicable to the proposal./Prière de sélectionner une option pour donner votre évaluation de l'intégration du sexe comme variable biologique en tant que point fort ou point faible de la proposition, ou en tant qu'élément non applicable à la proposition.

- (●) **Strength/Point fort**
- ( ) **Weakness/Point faible**
- ( ) **Not applicable/Non applicable**

Please indicate your appraisal of the integration of gender as a socio-cultural determinant of health as a strength, weakness, or not applicable to the proposal./Prière de sélectionner une option pour donner votre évaluation de l'intégration du genre comme déterminant socioculturel de la santé en tant que point fort ou point faible de la proposition, ou en tant qu'élément non applicable à la proposition.

- (●) **Strength/Point fort**
- ( ) **Weakness/Point faible**
- ( ) **Not applicable/Non applicable**

**Review Type / Type d'évaluation:**	Reviewer 3 / Évaluateur 3
**Name of Applicant / Nom du chercheur:**	Cassidy, Christine
**Application No. / Numéro de demande:**	526945
**Agency / Agence:**	CIHR/IRSC
**Competition / Concours:**	Project Grant/Subvention Projet
**Committee / Comité:**	Knowledge Translation Research/Recherche sur l'application des connaissances
**Title / Titre:**	I-STiCK: Implementing and Sustaining Transition in Care Innovations for Youth using Knowledge Translation Interventions

Sex and/or Gender Considerations/Notions de sexe et/ou de genre:

Well discussed for the team but less relevant for the implementation strategies.
